# The L7Ae protein binds to two kink-turns in the *Pyrococcus furiosus* RNase P RNA

**DOI:** 10.1093/nar/gku994

**Published:** 2014-10-31

**Authors:** Stella M. Lai, Lien B. Lai, Mark P. Foster, Venkat Gopalan

**Affiliations:** Department of Chemistry & Biochemistry, Center for RNA Biology, The Ohio State University, Columbus, OH 43210, USA

## Abstract

The RNA-binding protein L7Ae, known for its role in translation (as part of ribosomes) and RNA modification (as part of sn/oRNPs), has also been identified as a subunit of archaeal RNase P, a ribonucleoprotein complex that employs an RNA catalyst for the Mg^2+^-dependent 5′ maturation of tRNAs. To better understand the assembly and catalysis of archaeal RNase P, we used a site-specific hydroxyl radical-mediated footprinting strategy to pinpoint the binding sites of *Pyrococcus furiosus* (*Pfu*) L7Ae on its cognate RNase P RNA (RPR). L7Ae derivatives with single-Cys substitutions at residues in the predicted RNA-binding interface (K42C/C71V, R46C/C71V, V95C/C71V) were modified with an iron complex of EDTA-2-aminoethyl 2-pyridyl disulfide. Upon addition of hydrogen peroxide and ascorbate, these L7Ae-tethered nucleases were expected to cleave the RPR at nucleotides proximal to the EDTA-Fe–modified residues. Indeed, footprinting experiments with an enzyme assembled with the *Pfu* RPR and five protein cofactors (POP5, RPP21, RPP29, RPP30 and L7Ae–EDTA-Fe) revealed specific RNA cleavages, localizing the binding sites of L7Ae to the RPR's catalytic and specificity domains. These results support the presence of two kink-turns, the structural motifs recognized by L7Ae, in distinct functional domains of the RPR and suggest testable mechanisms by which L7Ae contributes to RNase P catalysis.

## INTRODUCTION

The maturation of nascent tRNAs requires multiple processing and modification steps. The Mg^2+^-dependent removal of the 5′ leader of precursor tRNAs (pre-tRNAs) is catalyzed by RNase P, an enzyme remarkable for the diversity of its subunit composition (1–3). While an RNA-free, protein-alone variant has been reported in some eukaryotes (4–6), the ribonucleoprotein (RNP) form is found in all domains of life. A catalytic RNase P RNA (RPR) is aided by a single RNase P protein (RPP) in bacteria and by as many as 10 RPPs in eukaryotes (1–3); archaeal RNase P is intermediate in complexity, with one RPR and up to five RPPs (1–3). This compositional variability in RNase P provides a model system for understanding how the function of an evolutionarily conserved ribozyme might be fine-tuned by different suites of protein cofactors.

By simultaneously binding the pre-tRNA leader and interacting with conserved nucleotides (nts) in the RPR, the bacterial RPP enhances the RPR's affinity for pre-tRNA and catalytically relevant Mg^2+^, thereby increasing the rate of pre-tRNA cleavage (7–10). In contrast, the roles of the multiple archaeal and eukaryotic RPPs, which make up ∼50% and ∼70%, respectively, of the molecular masses of their native enzymes (2), are not fully clear. To uncover the function of these protein subunits, archaeal RNase P has been used as an experimental proxy for the eukaryotic variant, which has not been reconstituted *in vitro*.

Human and yeast RNase P were purified to near homogeneity and their respective RPPs identified (11–13). Subsequently, four archaeal RPPs (POP5, RPP21, RPP29 and RPP30) were recognized based on homology to these eukaryotic RPPs and experimentally confirmed by their presence in partially purified native *Methanothermobacter thermautotrophicus* (*Mth*) RNase P (14). Additional evidence that these RPPs are indeed RNase P subunits came from their ability to aid RPR catalysis in *in vitro* reconstitution assays of both type A and type M euryarchaeal RNase P (15–20), which are classified based on the secondary structure of the RPR (21). The majority of euryarchaeal RPR variants are classified as type A [e.g. *Mth*, *Pyrococcus horikoshii* (*Pho*), *Pyrococcus furiosus* (*Pfu*)], based on their resemblance to the bacterial type A (Ancestral) RPRs and their catalytic activity *in vitro* in the absence of RPPs (21). The remaining variants categorized as type M RPRs [mostly from *Methanococcales*, e.g. *Methanocaldococcus jannaschii* (*Mja*) and *Methanococcus maripaludis* (*Mma*)] resemble the eukaryotic RPRs, and have been shown to be active *in vitro* in the presence of their RPPs or when a pre-tRNA is provided *in cis* (18,20,21).

**Figure 1.**
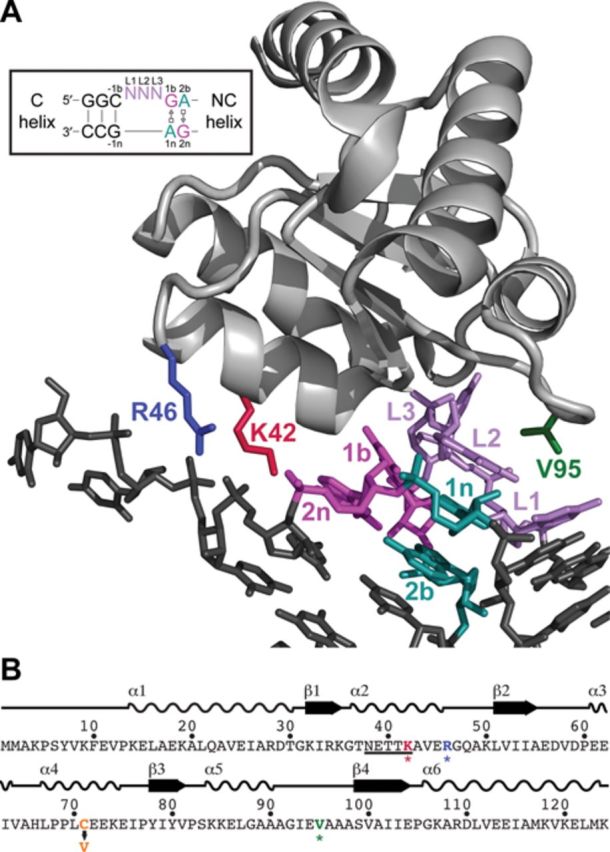
Residues selected for Cys substitution in *Pfu* L7Ae. (A) Tertiary structure of *Pfu* L7Ae in complex with an RNA [PDB: 3NVI (60)]. The positions where single-Cys residues were introduced are highlighted; this color scheme is used throughout the paper. Inset shows a standard K-turn motif, with canonical (C) and non-canonical (NC) helices shown (28); the two sheared G•A bps as well as the 3-nt bulge are color-coded in the inset and the crystal structure. (B) Amino acid sequence of *Pfu* L7Ae with its secondary structural elements indicated. Mutation of the native Cys to Val is noted while asterisks mark sites of single-Cys substitution and subsequent EPD-Fe modification.

Enzymes reconstituted *in vitro* from the four recombinant archaeal RPPs and the RPR were found to be less active and have a lower temperature optimum than native enzymes, prompting the hypothesis that archaeal RNase P may associate with additional RPPs *in vivo* (17,20,22). The L7Ae protein (∼14 kDa), which exhibits ∼25% identity to human RPP38 (∼35 kDa) (22–25), was tested as a possible fifth RPP and found to increase the temperature optimum of *in vitro* reconstituted *Pho* RNase P (22). Subsequently, the association of L7Ae with *Mma* RNase P was established by western analysis of fractions containing the partially purified native enzyme (20). Moreover, addition of *Mma* L7Ae to an enzyme assembled *in vitro* with *Mma* RPR, POP5, RPP21, RPP29 and RPP30 brings the optimal reaction temperature and *k*_cat_*/K*_M_ for pre-tRNA cleavage close to those observed with the native enzyme (20).L7Ae binds kink-turns (K-turns), which are widespread RNA structural motifs typified by a 5′ canonical (C) helix with standard Watson–Crick base pairs (bps), a 3-nt bulge and a 3′ non-canonical (NC) helix with two consecutive sheared G•A bps (Figure 1, inset) (26–33). The defining feature of the K-turn motif is a protein- and Mg^2+^-stabilized sharp kink that produces a 50°–60° angle between the two helices of the RNA, enabling RNA and protein interactions that would otherwise not be possible (28,33). While *in vitro* reconstitution experiments show that L7Ae is an important contributor to RNase P catalysis (20,22), identifying its RPR binding sites is critical for determining its mechanism of action. Given the diversity of euryarchaeal RPRs, it is also important to compare the interactions between L7Ae and type A or type M RPR variants to help elucidate whether L7Ae plays different functional roles in distinct euryarchaeal RNase P enzymes.

Our current understanding of L7Ae binding sites in archaeal RPRs is based on studies of *Pho* (type A) and *Mma* (type M) RNase P. Gel-shift assays with deletion mutants of *Pho* RPR identified two putative binding sites in the P12 and P15–16 regions (22), and subsequent studies with isolated RPR fragments sought to identify possible structural elements for L7Ae binding (34) (note: paired regions in the RPR are labeled as P1, P2, etc.). However, characterization of L7Ae-RPR binding interactions in *Pho* RNase P is inconclusive due to inconsistencies in the results to date. First, in contrast to earlier findings from gel-shift analyses, isothermal titration calorimetric (ITC) studies showed that an isolated P15–16 fragment failed to bind L7Ae (34). Second, these ITC studies also revealed an equimolar ratio of *Pho* RPR to L7Ae, even though an isolated P12 fragment bound two copies of the protein (34). Finally, deletion of the putative L7Ae binding site in P12 did not change the activity of *Pho* RNase P reconstituted with or without L7Ae (35). Therefore, firm experimental support for K-turns in type A RPRs is currently lacking. Meanwhile, examination of 10 different type M RPRs for conserved L7Ae binding sites led to the identification of a putative, non-consensus K-turn-like motif in the P12 region (20). Site-directed mutagenesis of this structural motif in the *Mma* RPR, followed by cleavage assays and footprinting experiments, was used to validate this K-turn-like motif as an L7Ae recognition site (20).

Despite evidence of a K-turn in the *Mma* (type M) RPR, such corroboration is still lacking in a type A RPR, and definitive mapping of L7Ae binding sites is necessary to understand its functional contributions to type A RNase P. For example, since P15–16 is a structural element in type A RPRs that plays a role in pre-tRNA and Mg^2+^ binding (36–38), unambiguous mapping of L7Ae binding sites (if any) in this region would gain additional significance. Moreover, the recent appreciation of motifs that depart from the standard K-turns, including the possibility of K-turn nts being distal in the RNA sequence, motivates the characterization of K-turn variants that allows for an expanded understanding of the role of L7Ae and its homologs (28,39). Experimental strategies that complement computational tools are therefore necessary to uncover such non-standard K-turns, as in the case of *Mma* RPR. In this study, following development of a stringent method to purify L7Ae free of nucleic acids, we used a site-specific hydroxyl radical (OH^•^)-mediated footprinting strategy (40–43) to identify the binding sites of *Pfu* L7Ae on its cognate RPR.

## MATERIALS AND METHODS

### Mutagenesis and purification of *Pfu* L7Ae derivatives

Three single-Cys derivatives (K42C/C71V, R46C/C71V and V95C/C71V) of *P. furiosus* DSM 3638 L7Ae (PF1367) were generated using polymerase chain reaction-based site-directed mutagenesis. Each L7Ae derivative was overexpressed in *Escherichia coli* and purified free of nucleic acids to near homogeneity by sequential use of anion-exchange, hydrophobic and reversed-phase chromatography. For additional details, refer to Supplementary Information, Supplementary Table S1 and Supplementary Figure S1.

### Modification of L7Ae with EDTA-2-aminoethyl 2-pyridyl disulfide-Fe (EPD-Fe)

Purified single-Cys derivatives of L7Ae were dialyzed against Buffer F (10 mM Tris-HCl, pH 8) supplemented with 5 mM DTT to ensure that the Cys residue in each derivative was fully reduced and available for modification. EPD (Toronto Research Chemicals, Toronto, Canada) was charged with equimolar FeCl_3_ for 15 min at 25°C before the resultant EPD-Fe complex was added in a 10-fold molar excess to L7Ae and incubated for 15 min at 25°C (Supplementary Figure S2). The EDTA-Fe–modified L7Ae samples were separated from the unreacted EPD-Fe complex and 2-pyridylthiol product using a MicroSpin G-25 Sephadex column (GE Healthcare). Successful modification of each L7Ae derivative was then verified using matrix-assisted laser desorption/ionization-time of flight mass spectrometry (Bruker Daltonics Microflex LT; Supplementary Table S2).

### RNase P activity assays

*Pfu* RPR and the two binary *Pfu* RPP complexes (POP5•RPP30 and RPP21•RPP29) were prepared as described elsewhere (17,19). To fold the RNA, 40 μM *in vitro*-transcribed *Pfu* RPR was incubated in water for 50 min at 50°C and 10 min at 37°C before adding an equal volume of 2× assay buffer (100 mM HEPES-KOH, pH 8; 1.6 M NH_4_OAc; 10 mM MgCl_2_) and incubating for an additional 30 min at 37°C (17,19). To assemble the *Pfu* RNase P enzyme, 10 nM folded RPR was pre-incubated with 100 nM each of POP5•RPP30, RPP21•RPP29 and either an unmodified or modified L7Ae derivative in 1× assay buffer for 10 min at 55°C. Previous biochemical studies showed that the RPPs function as binary complexes, and that a 10-fold excess of RPPs over RPR promoted optimal complex formation (17,19). The cleavage assay was then initiated by the addition of 500 nM pre-warmed *E. coli* pre-tRNA^Tyr^ (17), a trace amount of which was internally labeled with α-^32^P-GTP (∼10 000 dpm/reaction). For initial velocity measurements, five aliquots were withdrawn at 15-s intervals and immediately quenched in two volumes of urea dye [7 M urea, 1 mM EDTA, 0.05% (w/v) xylene cyanol, 0.05% (w/v) bromophenol blue, 10% (v/v) phenol]. Reaction products were separated on 8% (w/v) polyacrylamide/7 M urea gels and visualized using a Typhoon (GE Healthcare) phosphorimager. ImageQuant (GE Healthcare) was used to assess the extent of pre-tRNA^Tyr^ cleavage.

### Footprinting experiments

*Pfu* RPR was folded as described for activity assays, but with a 2× footprinting buffer, which had 12 mM MgCl_2_ (instead of 10 mM). For each footprinting reaction, the *Pfu* RNase P enzyme was reconstituted for 10 min at 55°C in 50 μl containing 50 nM folded *Pfu* RPR and 500 nM each of POP5•RPP30, RPP21•RPP29 and either an unmodified or modified single-Cys L7Ae derivative. Partial RNase P assemblies were prepared in a similar fashion but with the inclusion of only the indicated proteins.

Optimal reaction conditions for footprinting were empirically determined. The OH^•^-mediated cleavages were initiated by adding 5.5 μl of a freshly prepared solution containing 1% (v/v) hydrogen peroxide (Sigma, H-1009) and 25 mM L-ascorbic acid (Sigma, A-5960). The reaction was incubated for an additional 2 min at 55°C before it was terminated by adding 6.2 μl of freshly prepared 0.2 M thiourea (a radical scavenger; Sigma, T-8656) and then quickly frozen in liquid nitrogen. Subsequently, the contents of each reaction were thawed and treated with 40 μg of Proteinase K (Roche) for 35 min at 65°C. Following phenol-chloroform and chloroform extractions, the RNA was precipitated with 2 μl of 20 mg/ml glycogen, 8 μl of 3 M sodium acetate (pH 5.2) and 200 μl of 100% (v/v) ethanol and washed once with 75% (v/v) ethanol. The dried RNA samples were resuspended in 10 μl of water in preparation for primer extension.

### Analysis of footprinted RNA

EDTA-Fe(II)–catalyzed scissions in the RNA backbone were detected by primer extension using appropriate ^32^P-labeled primers (Supplementary Table S3). The annealing mixture containing 5 μl of footprinted RNA (above), 500 μM dNTPs, 50 nM gene-specific primer (GSP) and a trace amount of 5′-^32^P-GSP (1–3 × 10^5^ dpm) was incubated for 3 min at 100°C before snap cooling on ice. Maxima H-Minus Reverse Transcriptase (Thermo Scientific), buffer and RNase inhibitor were then added per manufacturer's instructions to a final volume of 10 μl and the reaction incubated in a thermal cycler for 1 min at 42°C and 30 min at 50°C. After the addition of 0.5 μl of 4 N NaOH, the reaction was incubated for 5 min at 95°C and then quenched with 14.5 μl of acid stop mix [4:25 (v/v) mixture of 1 M unbuffered Tris-HCl and stop dye (85% formamide; 0.5× TBE; 50 mM EDTA, pH 8; 0.05% (w/v) bromophenol blue; 0.05% (w/v) xylene cyanol)] and stored at –20°C.

Reference DNA sequencing ladders were generated by reverse transcription largely as described above with a few alterations. The annealing mixture for each of the four sequencing reactions (A, C, G and T) contained 300 nM *in vitro* transcribed *Pfu* RPR, 500 μM of the respective ddNTP (Roche), 250 μM dNTPs, 50 nM GSP and a trace amount of 5′-^32^P-labeled GSP (5 × 10^4^ dpm). PrimeScript Reverse Transcriptase (Clontech), buffer and RNase inhibitor were then added as per manufacturer's instructions to a final volume of 10 μl. After incubation in a thermal cycler, samples were treated with 10 units of terminal transferase (New England Biolabs) for 30 min at 37°C before addition of 0.5 μl of 4 N NaOH and incubation for 5 min at 95°C. Samples were then quenched with 14.5 μl of acid stop mix and stored at –20°C.

**Figure 2.**
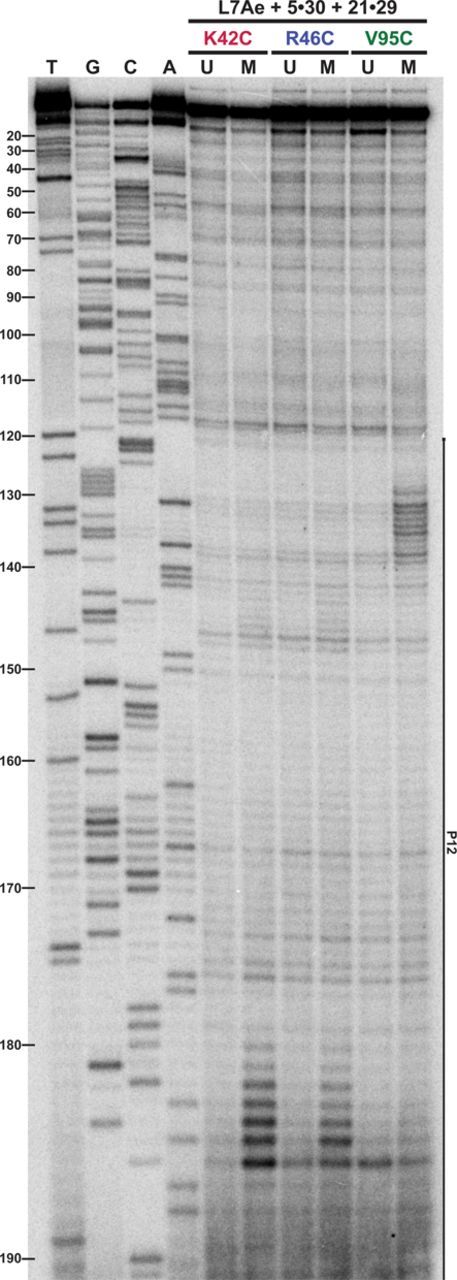
Mapping OH^•^-mediated cleavages of L7Ae–EDTA-Fe derivatives in the context of the 5-RPP enzyme on the P12 region of the *Pfu* RPR. RNA cleavage products were reverse transcribed using 5′-^32^P-PfuRPR-2R. U, unmodified; M, EDTA-Fe–modified.

**Figure 3.**
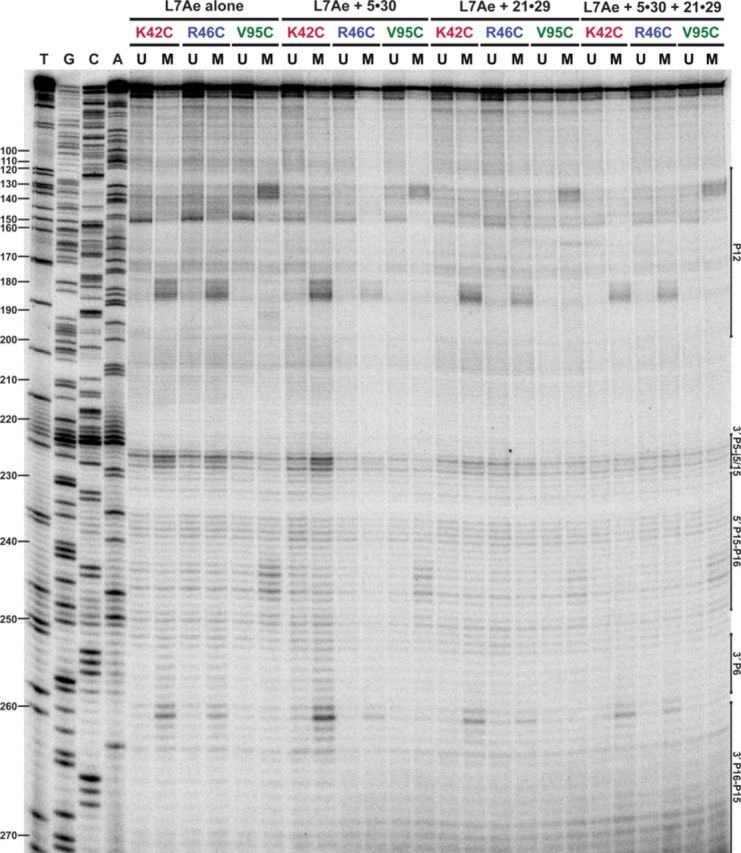
L7Ae binding sites on the *Pfu* RPR with and without other RPPs (POP5•RPP30 and RPP21•RPP29). RNA cleavage products were reverse transcribed using 5′-^32^P-PfuRPRj15/2-R. U, unmodified; M, EDTA-Fe–modified.

Products of the reverse transcription reactions were incubated for 20 min at 85°C before separation on a pre-warmed 8% (w/v) polyacrylamide/8 M urea sequencing gel (Sequagel, National Diagnostics); cDNA of footprinted RNAs were electrophoresed alongside their respective DNA sequencing ladders (Figures 2 and 3, and Supplementary Figure S3). The gels were fixed in a solution of 10% (v/v) glacial acetic acid and 10% (v/v) methanol, dried and scanned using a Typhoon phosphorimager. ImageQuant and Plot (http://plot1.micw.eu/) were used to compare the intensities of individual bands in each unmodified and modified reaction to determine the sites and extent of OH^•^-mediated cleavage (Supplementary Figure S4).

## RESULTS

### K42, R46 and V95 in *Pfu* L7Ae were substituted with Cys

The protein-RNA interactions observed in several crystallographic studies of L7Ae (and its homologs) bound to K-turn–containing RNA ligands (rRNA, C/D or H/ACA snoRNA) highlight the specific molecular recognition of this structural motif by L7Ae (e.g. Figure 1) (29–33). While some minor thematic variations exist, the characteristic protein-RNA interface seen in these structures reveals positions in *Pfu* L7Ae that, upon mutagenesis to Cys and modification with EPD-Fe, should reliably report on L7Ae binding sites in the *Pfu* RPR.*Pfu* L7Ae has an asymmetric electrostatic surface potential with a negatively and a positively charged face (30); the latter face includes two major structural elements favorable for RNA binding, a highly basic β-strand:turn:α-helix element and a short loop of hydrophobic residues (Figure 1) (28,32). Therefore, to map L7Ae-RPR interactions, interrogating probes should be positioned proximally to these RNA-binding structural motifs without disrupting RNP assembly.

The *trans* sugar-edge•Hoogsteen, sheared G•A bps at the 1b.1n and 2n.2b positions (Figure 1A, inset) are the signature feature of the K-turn motif (26). Not surprisingly, the K-turn–binding protein L7Ae has evolved a highly conserved N_38_E_39_xxK_42_ (*Pfu* L7Ae numbering) motif in α2 to interact with these conserved nts in the K-turn, specifically employing side-chain and backbone contacts to recognize G1b and G2n in the NC helix (Figure 1A, inset). Both N38 and E39 (as well as K42 in some cases) hydrogen bond with the major groove of these two conserved guanines (28,44). As expected, mutating the G•A bps results in loss of L7Ae binding and RNP assembly (45,46), and collectively mutating the three key residues of the NExxK motif also results in loss of function (20).

R46 shares the same helical face as the NExxK motif and is located one helical turn away from K42 at the C terminus of α2 (Figure 1A). These two basic residues contribute to the electrostatic stabilization of the L7Ae-RNA complex and may also hydrogen bond to the *proS* non-bridging oxygen atoms of the phosphate backbone [the 3n/4n and 4n/5n positions (28,32); Figure 1A, inset].

Situated on the opposite end of the same protein face as the NExxK motif (Figure 1A), V95 is part of an extended hydrophobic loop containing three key residues, I_93_E_94_V_95_ (*Pfu* L7Ae numbering), that are highly conserved among archaeal L7Ae homologs (31). These residues make close van der Waals contacts with the L1 and L2 nts of the K-turn [for examples, see (28,32)], with E94, the only charged amino acid in this loop, forming a more specific interaction through hydrogen bonding to L1 (32). V95 also forms one side of the hydrophobic pocket that binds the extruded L3 nt (30,31).

Based on the topography of the L7Ae–K-turn interface, K42, R46 and V95 in *Pfu* L7Ae were chosen for Cys substitution and EPD-Fe modification. While the first two residues in the N_38_E_39_xxK_42_ motif are essential for recognizing the sheared G•A bps, the less critical role of K42 suggests that it could be mutated and modified without disrupting RNP assembly, especially since R46 would still provide some electrostatic stabilization in that region in the absence of K42. A similar rationale applied to the choice of R46, given its proximity to K42. The hydrophobic pocket that envelops the K-turn's bulged loop might be able to accommodate an EDTA-Fe covalently attached at position 95, which would generate a footprint distinct from positions 42 and 46. Therefore, EDTA-Fe tethered to Cys residues at positions 42, 46 and 95 in *Pfu* L7Ae were expected to lead to OH^•^-mediated cleavages at or flanking the two G•A bps in any K-turn present in the RNA, thereby revealing this structural motif's location in the *Pfu* RPR. Toward this goal, three single-Cys substitutions (K42C, R46C and V95C) were introduced into a Cys-less *Pfu* L7Ae template (C71V); the native C71, which is partly buried, was mutated to Val to preserve the native fold and hydrophobic core of the protein.

### *Pfu* L7Ae derivatives were purified free of nucleic acids, modified with EPD-Fe and confirmed to be functionally active

A three-column procedure was used to purify all four *Pfu* L7Ae derivatives used in this study (Supplementary Figure S1A; see Supplementary Information). Given a predicted pI of 5.35 (http://web.expasy.org/protparam/), L7Ae was first subjected to anion-exchange chromatography before co-eluting protein contaminants were removed using a Phenyl Sepharose hydrophobic matrix. Although L7Ae appeared homogeneous as assessed by sodium dodecyl sulfate-polyacrylamide gel electrophoresis after these two chromatographic steps (Supplementary Figure S1B), its Abs_260/280_ ratio revealed that it remained largely bound to nucleic acids, necessitating a final stringent purification using reversed-phase high pressure liquid chromatography. Even after this final purification step, the Abs_260/280_ ratio of a few peak fractions suggested that they still contained L7Ae bound to nucleic acids, so only fractions with an Abs_260/280_ ratio of ∼0.5 were pooled and used for RNA footprinting analyses.

Purified single-Cys L7Ae derivatives (K42C/C71V, R46C/C71V and V95C/C71V) were then modified with EPD-Fe (Supplementary Figure S2). EPD was charged with iron and the resulting EPD-Fe complex reacted with the single surface-exposed Cys residue in L7Ae to attach an EDTA-Fe moiety and render each derivative a site-specific chemical nuclease. To ascertain whether EDTA-Fe had been successfully tethered to each L7Ae derivative, mass spectrometry was used to determine the molecular masses of each protein before and after modification. The observed masses were consistent with the expected values (Supplementary Table S2), and confirmed the identity and subsequent modification of the purified proteins.

To ensure that footprinting experiments were performed on functional RNase P complexes, each unmodified and EDTA-Fe–modified L7Ae derivative was tested for its ability to support RNase P activity. These functional assays were essential, as it is difficult to predict *a priori* which locations in L7Ae, upon attachment of EDTA-Fe, might disrupt assembly with the RNase P complex. *Pfu* RNase P has previously been reconstituted with *Pfu* RPR and two binary RPP complexes (POP5•RPP30 and RPP21•RPP29) to generate an enzyme that requires 30 mM Mg^2+^ for optimal activity (17). To better appreciate changes in activity due to mutation and modification of L7Ae, we established assay conditions that would discriminate between the activities of the 5-RPP and 4-RPP RNase P complexes (i.e. RPR + POP5•RPP30 + RPP21•RPP29 with or without L7Ae). In the presence of 5 mM Mg^2+^, the 5-RPP complex cleaves *E. coli* pre-tRNA^Tyr^ at a rate of 6–10 min^−1^ (Table 1) while the 4-RPP complex is minimally active (∼0.4 min^−1^).

**Table 1.** Activity of *Pfu* RNase P enzymes reconstituted with unmodified or modified L7Ae derivatives

Protein	Turnover (min^−1^)
Wild type	6.38 ± 0.70
C71V	9.95 ± 0.84
K42C	10.8 ± 0.48
K42C–EDTA-Fe	10.8 ± 0.39
R46C	12.3 ± 1.18
R46C–EDTA-Fe	8.88 ± 0.75
V95C	12.4 ± 0.63
V95C–EDTA-Fe	11.7 ± 0.64

Turnover number of *Pfu* RNase P enzymes reconstituted with the RPR + POP5•RPP30 + RPP21•RPP29 + either an unmodified or modified L7Ae derivative was determined as described in Materials and Methods. Enzymes reconstituted with L7Ae C71V were more active than those with the wild type. The mean and standard deviation in each case were calculated from three independent experiments.

Multiple-turnover assays in the presence of 5 mM MgCl_2_ with each of the unmodified and EDTA-Fe-modified single-Cys L7Ae derivatives showed activity similar to or better than the C71V ‘parental’ reference, which in turn was more active than the wild type (Table 1). The covalent tethering of EDTA-Fe to R46C and V95C causes only a 28% and 6% decrease in activity, respectively, relative to their unmodified counterparts; such modest decreases may be due to the addition of a bulky group in the RNA-binding interface. Modification of K42C, however, did not affect activity. Overall, the results from these activity assays showed that neither the single-Cys substitutions nor the tethering of EDTA-Fe to these Cys residues significantly perturbed the function of L7Ae in the context of the *Pfu* RNase P enzyme (Table 1).

### *Pfu* L7Ae footprints at two exclusive sites on the RPR

OH^•^-mediated footprinting requires the addition of ascorbate and hydrogen peroxide. EDTA-Fe(III), which is covalently tethered to a Cys residue, is reduced to EDTA-Fe(II) by ascorbate (40). Hydrogen peroxide then reacts with Fe(II) to generate Fe(III) and a highly reactive OH^•^, which induces the oxidative degradation of proximal ribose units, thereby breaking the phosphodiester backbone. Fe(III), thus generated by the Fenton reaction, is again reduced to Fe(II) by ascorbate, allowing another round of OH^•^ generation. Due to the short lifetime of the radical in aqueous solution, the OH^•^-mediated attack on solvent-exposed ribose units can only occur within ∼10 Å of the Fe atom (40). With the Fe atom positioned only ∼14 Å away from the C^α^ in the tethered Cys residue, the OH^•^-mediated scissions help localize the protein's binding sites on its RNA ligand.

For each *Pfu* L7Ae derivative, two footprinting reactions were performed: one with the unmodified protein and the other with the EDTA-Fe–modified version. In both reactions, L7Ae was assembled with *Pfu* RPR, POP5•RPP30 and RPP21•RPP29 to form the respective RNase P enzyme. The resulting OH^•^-mediated cleavage products were reverse transcribed with four different 5′-radiolabeled primers, complementary to different regions of the *Pfu* RPR (Supplementary Table S3), to scan for cleavages along its entire length (330 nts); nine different primers were tested before choosing the four that yielded the most consistent cDNA profiles and the fewest false stops, which are important considerations for GC-rich RNAs, such as the *Pfu* RPR. These cDNA products were then separated on sequencing gels and visualized by phosphorimaging. Comparing the band intensities of the two footprinting reactions (unmodified versus modified) in these primer extension assays, either by visual examination or image quantitation (Supplementary Figure S4), provides a direct readout of the sites in the *Pfu* RPR that are cleaved due to their proximity to Cys-tethered EDTA-Fe.

**Figure 4.**
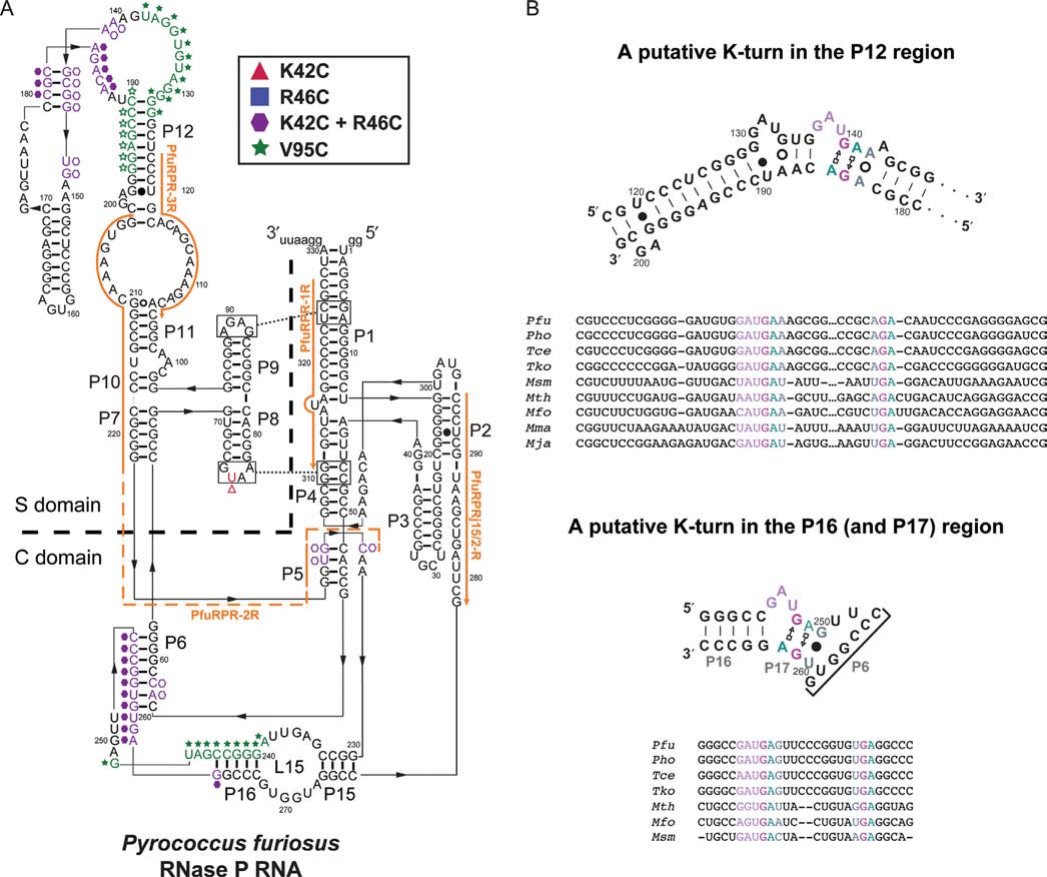
(A) The OH^•^-mediated cleavages of *Pfu* RPR promoted by *Pfu* L7Ae K42C–, R46C– and V95C–EDTA-Fe (in the absence or presence of other RPPs) are summarized on the secondary structure using filled triangles, squares and stars, respectively, and the same color scheme as in Figure 1. Overlapping K42C and R46C footprints are indicated using purple hexagons. Open triangles, hexagons and stars denote weak footprints in the P5, P6, L8 and P12 regions (see text for additional details). The catalytic (C) and substrate specificity (S) domains are also marked. The secondary structure of the *Pfu* RPR was modeled using as template a bacterial RPR whose high-resolution tertiary structure is available (Supplementary Figure S6). (B) Putative K-turns in the P12 and P16–17 regions of the RPR from *Pfu* (secondary structures shown) and its close relatives. In addition to our footprinting data, the assignment of K-turns in the *Pfu* RPR is supported by sequence alignment of P12 and P16 regions from six other euryarchaeal type A RPRs belonging to *Thermococcales* and *Methanobacteriales*. In the top panel, the sequence alignment also includes two RPRs from *Methanococcales* to show broader conservation of the P12 K-turn in type A and M RPRs. *Mfo*, *Methanobacter formicicum*; *Msm*, *Methanobrevibacter smithii*; *Tce*, *Thermococcus celer*; *Tko*, *Thermococcus kodakaraensis*.

Cleavages generated by each L7Ae derivative were primarily localized to two distinct regions of the RPR: the P12 and P16 regions. The OH^•^-mediated cleavages were highly reproducible and, in each case, verified by at least three independent replicates. Moreover, the four primers used in this study (Figure 4A and Supplementary Table S3) allow for overlapping scans of each section of the RPR and provide another robust measure for the reproducibility of the footprinting data; for example, primers PfuRPRj15/2-R and PfuRPR-2R independently confirm footprints in the P12 region and likewise, PfuRPR-1R (Supplementary Figure S3A) and PfuRPRj15/2-R for the P16 region. As expected from their proximity in the tertiary structure of L7Ae, K42C– and R46C–EDTA-Fe caused OH^•^-mediated cleavages at similar and overlapping sites in the RPR, with R46C–EDTA-Fe giving relatively weaker hits, consistent with its modest decrease in activity upon modification.

In the P12 region, prominent cleavages by K42C– and R46C–EDTA-Fe were observed at positions 180–187 (Figure 2), with the more intense hits evident at positions 182–186, coinciding with A1n and G2n of the G•A bps in a putative K-turn (Figure 4B). This pattern is consistent with the crystal structures of L7Ae-RNA complexes, which show that K42 and R46 interact with the 3n/4n and 4n/5n backbone phosphates of the RNA ligand (28,32). The recurring pattern within each set of footprints, of weaker secondary cleavages immediately flanking the primary cleavage sites, is consistent with diffusion of OH^•^ from the site of generation (40,43). Located on the opposite end of the RNA-binding surface from positions 42 and 46, V95C–EDTA-Fe promoted unique cleavages that map to the same vicinity in P12 as K42C– and R46C–EDTA-Fe but on the opposite strand: strong cleavages were observed at positions 130–134 with weaker ones at 126–129 and 135–138 (Figure 2). These hits encompass the C helix and the 3-nt bulge of a putative K-turn (Figure 4B), consistent with V95 binding the K-turn bulge in crystal structures.

In the P16 region, strong cleavages by K42C– and R46C–EDTA-Fe at positions 258 and 259 are accompanied by weaker ones at 253–257 and 260–263 (Figure 3), which include both the A1n and G2n of the G•A bps in a putative K-turn. Nucleotides 57 and 58 in the 5′ strand of the P6 pseudoknot were also cleaved by K42C– and R46C–EDTA-Fe (Supplementary Figure S3B), consistent with cleavages at 257 and 258 (which base pair with 58 and 57, respectively) in the 3′ strand of this pseudoknot (Figures 3 and 4A). The P6 helix is likely to be contiguous with P16 (or to be precise, P17, which is formed in the presence of a K-turn; Figure 4B), based on comparisons with the bacterial type A RPR high-resolution structure (47). With V95C–EDTA-Fe, hits of varying intensities are observed from 239–248, likely due to differences in solvent accessibility (Figure 3). These cleavages, like those in P12, map to the 5′ strand of the C helix and the 3-nt bulge of a putative K-turn (Figure 4B).

The above-mentioned P12 and P16 footprints are clearly evident from even a visual inspection of the footprinting data. Indeed, image quantitation confirms that the main hits in a suite of strong neighboring cleavages are ≥2-fold more intense than cleavages in the unmodified reaction (Supplementary Figure S4). Other cleavages, while still discernible, are below this threshold. To illustrate, K42C– and R46C–EDTA-Fe footprint weakly at positions 141–148, and V95C–EDTA-Fe at positions 190–196 (Figures 2 and 3); these hits in P12 are located on the opposite strand immediately across from the strong cleavages described above for each of the modified Cys residues (open versus filled symbols, respectively, in Figure 4A). While the strong cleavages in P12 and P16 are in remarkable agreement (e.g. A1n and G2n of the G•A bps hit by K42C– and R46C–EDTA-Fe, and L3 of the K-turn hit by V95C–EDTA-Fe), the weaker hits in P12 extend the footprint further than that observed in P16 (Figure 4A). Either differences in the local architecture (such as a fold-back of P12, see below) or another weaker binding site in P12 may have resulted in a larger footprint for L7Ae in P12 compared to P16.

Another weak but reproducible footprint was also observed with K42C–EDTA-Fe: a unique footprint in the terminal loop of P8 at position 75 (Supplementary Figure S3B). The proximity of this region to those with strong hits in either P12 or P16 in the tertiary structure of the RPR remains to be established.

### L7Ae footprints on the *Pfu* RPR are the same even in the absence of other RPPs

Given previous studies with *Pfu* RNase P that showed that the addition of POP5•RPP30 or RPP21•RPP29 to the *Pfu* RPR significantly increased its activity at lower substrate and Mg^2+^ concentrations (17), we tested whether L7Ae binding to the RPR is influenced by other RPPs. Footprinting experiments were performed with an L7Ae–EDTA-Fe derivative alone or with either POP5•RPP30 or RPP21•RPP29. Regardless of whether the other RPPs were present, the L7Ae footprints remained essentially the same (Figure 3), although some neighboring hits were more apparent in the L7Ae-alone reactions than in the other reactions due to modest variations in signal intensity. For example, when the RPR is footprinted with just L7Ae instead of all five RPPs, P16 footprints from K42C–, R46C– and V95C–EDTA-Fe comprised some additional weak hits (e.g. 253–263 instead of 258–263; Figure 3). Similarly, both K42C– and R46C–EDTA-Fe showed modest enhancements in cleavage of the 3′ strand of P5 at positions 225–227 (Figure 3), which were not apparent after addition of the other RPPs (Figure 3 and Supplementary Figure S3). Such differences may reflect either metastable regions in the RPR in the absence of the other RPPs or protection of regions adjacent to the L7Ae binding sites by the other RPPs. Regardless, the strong agreement between L7Ae footprints with and without other RPPs lends additional confidence in assigning the L7Ae binding sites on the RPR.

### The two putative K-turns in *Pfu* RPR are conserved in close relatives

Based on the highly reproducible L7Ae footprints and the predicted secondary structure of the *Pfu* RPR (Figure 4A), it is possible to assign putative K-turns in both the P12 and P16 regions (Figure 4B). These K-turns in the *Pfu* RPR are likely conserved as they are also found in nearly identical locations in the sequences and secondary structures of other type A RPRs (Figure 4B). In fact, the location of the K-turn in the P12 region of the *Pfu* RPR mirrors those previously reported in archaeal type M RPRs (20; type M RPRs do not have P15–16) as well as eukaryotic RPRs and RNase MRP RNAs (which are evolutionarily related to RPRs) (48,49).

## DISCUSSION

Footprinting experiments using *Pfu* RNase P enzymes assembled with L7Ae K42C–, R46C– and V95C–EDTA-Fe revealed specific RPR cleavages, localizing the binding sites of L7Ae to two disparate regions, P12 and P16. Since L7Ae specifically recognizes and binds K-turns, the sequences in these regions were subsequently examined and putative K-turns identified (Figure 4B). In high-resolution structures of L7Ae bound to different K-turns (e.g. Figure 1), K42 and R46 are proximal to the 3′ strand of the NC helix, including the G•A bps, while V95 lies close to the 3-nt bulge and the 5′ strand of the C helix. In the P12 and P16 regions of the *Pfu* RPR, there are two similar sets of L7Ae footprints that are consistent with this binding interface: K42C– and R46C–EDTA-Fe led to cleavages at and 3′ to the putative G•A bps while V95C–EDTA-Fe resulted in hits 5′ to the G•A bps, including the 3-nt bulge. Moreover, these footprints are consistent with the diffusion radius of the OH^•^ and the intermolecular distances measured from the C^α^ of residues 42, 46 and 95 in L7Ae to the phosphate backbone in or adjacent to the K-turn (see representative examples in Supplementary Figure S5).

While the footprinting data suggest a stoichiometry of two L7Ae copies bound to one *Pfu* RPR (one each to P12 and P16), the extended footprints in P12 may reflect a lower affinity site capable of binding an additional copy of L7Ae. Consistent with this possibility, ITC studies with a nearly 10-fold higher concentration of L7Ae than that used here had implicated binding of two L7Ae copies to an isolated P12 fragment from *Pho* RPR (34). Further studies (e.g. chemical footprinting with dimethylsulfate, native mass spectrometry to confirm L7Ae stoichiometry, tertiary structure determination) are needed to confirm if there are indeed two K-turns in P12, including one that is possibly a non-standard variant.

Clues regarding the role of L7Ae in RNase P function emerge from considering its footprints in P12 [specificity (S) domain] and P16 [catalytic (C) domain] with and without the other RPPs. Our results indicate that the L7Ae-RPR interactions observed in the context of the fully assembled RNase P enzyme are the same without the other RPPs (Figure 3), and provide support for a scaffolding role for L7Ae during RNase P assembly. There is growing evidence that archaeal RPRs, like their bacterial counterparts, have a modular structural composition: the S domain that recognizes the T stem-loop of the pre-tRNA and the C domain that can cleave the pre-tRNA while binding to its 5′ leader, acceptor stem and 3′ RCCA (17–19,50,51). Such a structural and functional divide in archaeal RPRs is facilitated by the RPPs – enzymatic (RNase T1 and V1) footprinting studies indicate that RPP21•RPP29 and POP5•RPP30, either alone or together, interact exclusively with the S and C domains, respectively (17,52). These binding sites correlate well with the functional effects elicited by each binary pair; single-turnover kinetic studies with *Mth* RNase P revealed that RPP21•RPP29 increases the apparent substrate affinity (*K*_S_) by 16-fold while POP5•RPP30 is solely responsible for enhancing the RPR's rate of cleavage by 60-fold (19). As a parallel then, the binding of *Pfu* L7Ae to the K-turns in the S and C domains may promote optimal binding and function of RPP21•RPP29 and POP5•RPP30, respectively, given the proximity of the two K-turns to the footprints of the two binary RPP complexes (17,52). Such a dependency would be consistent with a scaffolding role for L7Ae in the RPR's S and C domains.

In addition to its role in archaeal RNase P, L7Ae is an integral subunit of C/D box and H/ACA snoRNPs, which catalyze 2′-*O*-methylation and rRNA pseudo-uridylation, respectively (53,54). However, the L7Ae requirement in these RNPs is not the same. The C/D box snoRNP is not active without L7Ae because L7Ae binding drives the specificity and affinity for proteins that subsequently assemble into the final complex [(54), see (55) for a thematic variation with a *cis* substrate]. In contrast, addition of L7Ae enhances the activity of H/ACA snoRNP and RNase P. L7Ae may not be critical for initiating RNase P assembly under *in vitro* Mg^2+^ conditions (7.5–50 mM in previous studies) (15,17,20); however, at limiting intracellular Mg^2+^ concentrations (56), L7Ae likely stabilizes the K-turns in the S and C domains of the RPR to generate local structures with high electronegative density and shape/surface features that are suitable for binding to RPP21•RPP29 and POP5•RPP30, respectively. Such a critical role may not be readily discernible in *in vitro* assays with high concentrations of Mg^2+^ that could fulfill a similar function as L7Ae by alleviating charge repulsion and stabilizing K-turns that engender tertiary contacts and RPP interactions.

While the K-turn that we have identified in P16 of type A archaeal RPRs (Figure 4B) is not present in some bacterial counterparts, a new structural motif called the pK-turn was recently reported in the P16-P17 region of the *Thermotoga maritima* (*Tma*) RPR (57). While lacking the standard features of a canonical K-turn (i.e. no G•A bps or long-range hydrogen bonds between the two helices), the pK-turn supports a kinked geometry in the context of the RPR's tertiary structure (57). Substitution of the pK-turn in *Tma* RPR with a K-turn resulted in an active enzyme, albeit with only 20% activity as that of the wild type. This functional inter-changeability was interpreted as indicative of different structural solutions to introduce a kink in P16-P17 of bacterial and archaeal RPRs (57). The kink in P16–17 may be critical for forming the P6 pseudoknot and the Mg^2+^-bound L15, which enables direct base pairing with the 3′ RCCA of the pre-tRNA substrate (36). If indeed L7Ae promotes RPR–pre-tRNA interactions, it would represent an interesting similarity with H/ACA snoRNPs where L7Ae is believed to remodel the guide RNA for binding to an RNA substrate that undergoes pseudo-uridylation (58). Such remodeling also highlights how the scaffolding role of L7Ae might contribute directly to function.

The difference in the number of L7Ae binding sites in type A and type M RPRs (two versus one, respectively, due to the presence and absence of P16) has an interesting parallel in the ribosome. Cryo-electron microscopy-based studies recently reported that L7Ae, first identified as a large rRNA-associated subunit, has a second binding site on the small rRNA subunit and even a third site on the large rRNA (59). However, this third site appears to be missing in ribosomes of late branches of euryarchaea (e.g. *Methanococcales*). Thus, despite a unifying scaffolding role for L7Ae, the frequency of its use in this capacity may differ, even in homologous multi-subunit RNPs, reflecting the co-evolution of L7Ae with cognate RNA ligands and associated proteins. Genome editing may be used to help dissect the phenotypic consequences of such variability.

## SUMMARY

The ability of *Mma*, *Pfu* and *Pho* RPRs to bind RPP21•RPP29 and POP5•RPP30 to form functional enzymes without L7Ae (15,17,20) has made the specific role of L7Ae in RNase P catalysis difficult to appreciate. Results from our footprinting studies on *Pfu* RNase P now indicate that binding of L7Ae to K-turns in the S and C domains of the RPR may engender structural changes to allow productive interactions with RPP21•RPP29 and POP5•RPP30, respectively. Such a conceptual framework is reminiscent of the ability of L7Ae to remodel the H/ACA and C/D box guide RNAs for better binding to either target substrates or other protein subunits (54,58,60). Elucidating the L7Ae-mediated structural alterations in the *Pfu* RPR, especially under physiological Mg^2+^ concentrations, will be an important next step for determining its essential role in RNase P assembly and catalysis. The L7Ae derivatives that served as site-specific chemical nucleases in this study merit inclusion in the molecular toolkit for experimental validation of computationally identified novel K-turn variants, especially those formed by nts in distal parts of the same RNA (39).

## SUPPLEMENTARY DATA

Supplementary Data are available at NAR Online.

SUPPLEMENTARY DATA
