# Supplementary material for: The L7Ae protein binds to two kink-turns in the Pyrococcus furiosus RNase P RNA
Source: Nucleic Acids Res. 2014 Oct 31;42(21):13328–38. doi: 10.1093/nar/gku994 (PMC4245976; doi:10.1093/nar/gku994)
Supplement: SUPPLEMENTARY DATA [file supp_42_21_13328__index.html]

The L7Ae protein binds to two kink-turns in the Pyrococcus furiosus RNase P RNA — The L7Ae protein binds to two kink-turns in the Pyrococcus furiosus RNase P RNA — SUPPLEMENTARY DATA 

# The L7Ae protein binds to two kink-turns in the *Pyrococcus furiosus* RNase P RNA

## SUPPLEMENTARY DATA

**Files in this Data Supplement:**

- SUPPLEMENTARY DATA
