# Supplementary material for: The L7Ae protein binds to two kink-turns in the Pyrococcus furiosus RNase P RNA
Source: Nucleic Acids Res. 2014 Oct 31;42(21):13328–38. doi: 10.1093/nar/gku994 (PMC4245976; doi:10.1093/nar/gku994)
Supplement: SUPPLEMENTARY DATA [file supp_gku994_nar-01939-r-2014-File006.pdf]

SUPPLEMENTARY INFORMATION

The L7Ae protein binds to two distinct sites in the *Pyrococcus furiosus* RNase P RNA

Stella M. Lai, Lien B. Lai, Mark P. Foster*, and Venkat Gopalan*

Department of Chemistry & Biochemistry, Center for RNA Biology,

The Ohio State University, Columbus, OH 43210, USA

* Corresponding authors:

Mark P. Foster (Tel: 614-292-1377; Email: foster.281@osu.edu)

Venkat Gopalan (Tel: 614-292-1332; Email: gopalan.5@osu.edu)

MATERIAL AND METHODS

Site-directed mutagenesis of *Pfu* L7Ae

Three single-Cys-substituted derivatives (K42C/C71V, R46C/C71V, and V95C/C71V) of *Pyrococcus furiosus* DSM 3638 L7Ae (PF1367) were generated by PCR, using as template pET-33b–*Pfu* L7Ae C71V, which encodes a Cys-less L7Ae. Oligonucleotides were designed to introduce a single Cys codon at the desired location in the L7Ae gene (Table S1). Each mutagenesis entailed amplification of the entire plasmid by Phusion® High-Fidelity DNA Polymerase (Finnzymes) using a pair of 5'-phosphorylated primers that were oriented outward (back-to-back) and that included the codon to be mutated at the 5'-end of one of the primers (Table S1). These PCR products were circularized by ligation before electroporation into *Escherichia coli* DH5α cells. To confirm positive clones, plasmid DNA was isolated from individual transformants, screened using restriction digests (Table S1), and subjected to automated DNA sequencing at the OSU Plant-Microbe Genomics Facility.

Overexpression and purification of *Pfu* L7Ae

For overexpression of the L7Ae derivatives (wild type, C71V, K42C/C71V, R46C/C71V, and V95C/C71V), *E. coli* BL21 (DE3) Rosetta cells were transformed with the appropriate plasmids. A single colony was used to inoculate 5 ml of LB medium containing 35 μg/ml each of kanamycin and chloramphenicol and grown ~16 h at 37°C with shaking. This overnight culture was used to inoculate 500 ml of LB medium containing the same antibiotics and subsequently grown at 37°C with shaking. Protein overexpression was induced with 1 mM isopropyl-β-D-thio-galactoside (IPTG) at an OD_{600} ~ 0.6, and the culture was grown for an additional 4 h at 37°C with shaking. The cells were harvested by centrifugation and stored as a pellet at –80°C until use.

All L7Ae derivatives were purified without associated nucleic acids to near homogeneity by sequential use of anion-exchange, hydrophobic, and reversed-phase chromatography (Fig. S1). While the first two steps entailed use of an ÄKTA FPLC purifier (GE Healthcare), the third was performed using a Waters HPLC system.

Each 500-ml cell pellet (above) was resuspended in 45 ml of Buffer L (25 mM Tris-HCl, pH 8; 5 mM DTT; 0.1 mM PMSF) and sonicated for a total of 6 min on ice with a Vibra-Cell Ultrasonic Processor (GEX130) using a stepped 6-mm microtip and the following settings: 5 s ON (pulse), 2 s OFF (interval); setting 3; amplitude 50%. Following centrifugation (9,000 *g*; 15 min; 4°C), the supernatant was first incubated for 30 min at 25°C with 10 units of DNase I (Fermentas) and 10 mM $MgCl_2$, and then for 30 min at 80°C to precipitate a number of *E. coli* proteins, which were then removed by centrifugation at 30,000 *g* for 20 min at 4°C. The resulting supernatant was passed through a 0.22-μm bottle-top vacuum filter to yield the input for subsequent purification.

The input was then loaded on a pre-equilibrated 5-ml HiTrap Q Sepharose HP column (GE Healthcare) and eluted with a 0–0.8 M NaCl gradient in Buffer L supplemented with 1 mM EDTA. Fractions containing *Pfu* L7Ae, which eluted ~180 mM NaCl, were identified using SDS-PAGE and pooled. Based on a calculation of the total volume and average concentration of NaCl in the pooled sample, solid NaCl was added

to yield a final concentration of 3 M NaCl.

The Q Sepharose-purified protein sample was then filtered using a 0.45-μm syringe filter and loaded on a pre-equilibrated 1-ml HiTrap Phenyl Sepharose HP column (GE Healthcare). Unlike some contaminating proteins present in the Q Sepharose eluate, *Pfu* L7Ae does not bind to this hydrophobic matrix. The *Pfu* L7Ae-containing flow-through was then dialyzed against Buffer L at 25°C to remove NaCl in preparation for HPLC.

For the final purification step, the dialyzed sample was loaded on an analytical C4 reversed-phase column (Grace Vydac 214TP5414; 4.6 mm internal diameter and 150 mm length) and eluted at 1 ml/min with solvents A (99.9% water, 0.1% TFA) and B (99.9% acetonitrile, 0.1% TFA) using the following program: 5 ml of 5% B, 32.5 ml linear gradient of 5–95% B, and 2.5 ml of 5% B. The HPLC eluate was collected in 1-ml fractions, and peak fractions (~40-50% acetonitrile) were identified using the Abs_{220} and Abs_{280} profiles. These fractions were individually frozen in liquid nitrogen, lyophilized, resuspended in small volumes (50–100 μl, depending on the size of the lyophilized pellet) of Buffer R (20 mM sodium phosphate, pH 6.5; 6 M guanidinium hydrochloride), and used for absorbance measurements. To minimize nucleic acid contamination, only fractions with an $Abs_{260/280}$ ratio of ~0.5 were pooled and their total volume brought to 15 ml with additional Buffer R. Refolding of L7Ae was initiated by overnight dialysis against 3 L of Buffer M (10 mM Tris-HCl, pH 8; 10 mM KCl; 5 mM DTT), followed by dialysis against Buffer F (10 mM Tris-HCl, pH 8) prior to quantitation (by measuring Abs_{280}) and storage at –80°C.

Table S1
DNA oligonucleotides used for site-directed mutagenesis of *Pfu* L7Ae

Primer	Primer Sequence	(±) Restriction Enzyme Site
C71V-F	CCACCACTC**GTT**GAGGAGAAGGAGATTC	(-) AdeI/DraIII
C71V-R	AAGGTGTGCTACAATCTCTTCTGGATCAACATC	
K42C-F	**TGT**GCTGTAGAGAGAGGCCAAGC	(+) PciI
K42C-R	TGTGGTTTCATTTGTTCCCTTTCTAATC	
R46C-F	A**TGC**GGCCAAGCAAAGCTC	(+) BsmI
R46C-R	TCTACAGCCTTTGTGGTTTCATTTGTTC	
V95C-F	**TGC**GCAGCCGCAAGTG	(+) BsmI/FspI
V95C-R	TTCAATGCCAGCTGCAGCTC	

K42C, R46C, and V95C mutations were each obtained using as template *Pfu* L7Ae C71V, which was first generated using the wild type as template. The mutagenesis design ensured changes in the restriction pattern to facilitate rapid screening of mutant plasmids. In each primer (shown above in 5' to 3' orientation), boldface nucleotides indicate Cys-substituted codons while the underlined nucleotide indicates a neutral substitution to alter the restriction pattern. (-) and (+) signify loss and gain of the specified restriction enzyme site.

Table S2
Molecular masses of L7Ae derivatives before and after modification

Protein	Predicted mass (Da)	Observed mass (Da)
**C71V**	13,522	13,521
**K42C**	13,497	13,495
**K42C–EDTA-Fe**	13,899	13,895
**R46C**	13,469	13,468
**R46C–EDTA-Fe**	13,871	13,870
**V95C**	13,526	13,526
**V95C–EDTA-Fe**	13,928	13,923

Molecular masses were measured using matrix-assisted laser desorption/ionization-time of flight (MALDI-TOF) mass spectrometry to confirm successful modification. The observed mass of each protein was based on the singly-charged species observed in the mass spectrum. The EDTA-Fe modification adds 402 Da to the overall mass.

Table S3
DNA oligonucleotides used for primer extension

Primer	Primer Sequence	Nucleotides in the *Pfu* RPR
PfuRPR-1R	GCGAGGGGGCTATAGCC	327-311
PfuRPR-2R	GGACGGCCGTTTCACC	227-202
PfuRPR-3R	GGGACGTGTCGTTTCTGTG	123-105
PfuRPRj15/2-R	GGGAGCATTCGACTAAGC	295-278

These DNA oligonucleotides (shown above in 5' to 3' orientation), which are complementary to the indicated nucleotides in the *Pfu* RPR, were used for run-off reverse transcription of RNA cleavage products from $OH^{\bullet}$-mediated footprinting experiments. See Fig. 4A for the exact locations of the primers in the secondary structure of the *Pfu* RPR.

A

Transformation of *E. coli* BL21 (DE3) Rosetta expression cells with plasmids encoding single-Cys L7Ae derivatives

↓

Growth of 0.5 L culture to Abs_{600} ~ 0.6

↓

Induction: 1 mM IPTG, 4 h, 37°C

↓

Resuspension in Buffer L

↓

Sonication, centrifugation, and DNase I treatment (10 U) for 30 min at 25°C

↓

Incubation for 30 min at 80°C and centrifugation

↓

Application to a Q Sepharose column and elution with a 0-0.8 M NaCl gradient

↓

Addition of NaCl to 3 M and application to a Phenyl Sepharose column:
nearly pure L7Ae in the flow-through

↓

Reversed-phase HPLC using a C4 column **to remove tightly-bound nucleic acids**; collect all HPLC eluate in 1-ml fractions

↓

Resuspension of each lyophilized fraction in small volume to determine $Abs_{260/280}$ ratio; pool fractions with ratio ~0.5

↓

Refold protein

B

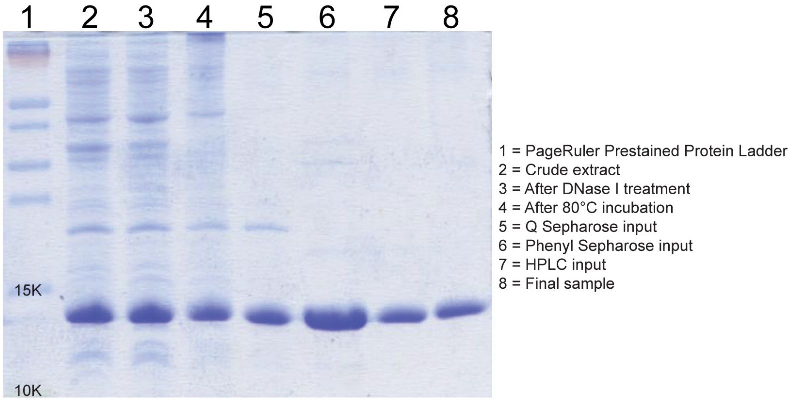

Figure S1. Optimized protein over-expression and purification of *Pfu* L7Ae. (A) Outline of the three-column purification procedure required to obtain nucleic acid-free L7Ae. (B) Representative SDS-PAGE of *Pfu* L7Ae purification. While the protein appears free of contamination following the Phenyl Sepharose purification step, the $Abs_{260/280}$ ratio indicates the presence of nucleic acids in the sample.

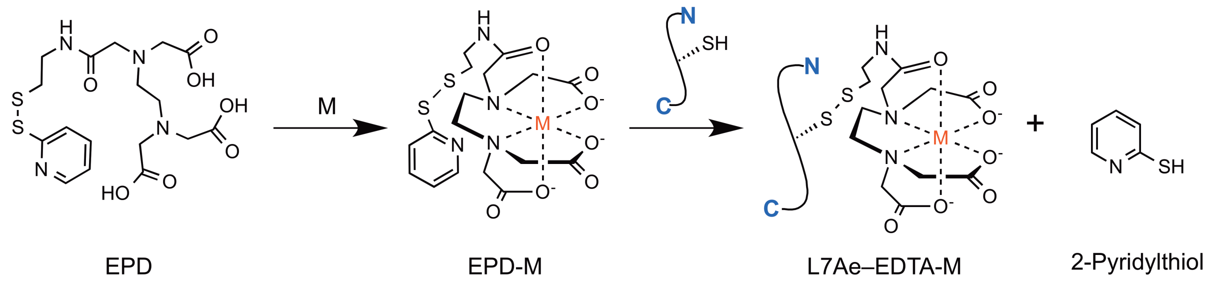

Figure S2. Modification of a single-Cys-substituted L7Ae derivative with EDTA-2-aminoethyl 2-pyridyl disulfide (EPD) charged with iron (M) to generate a site-specific nuclease (L7Ae–EDTA-M) for $OH^{\bullet}$-mediated footprinting. Figure adapted from ref. 1.

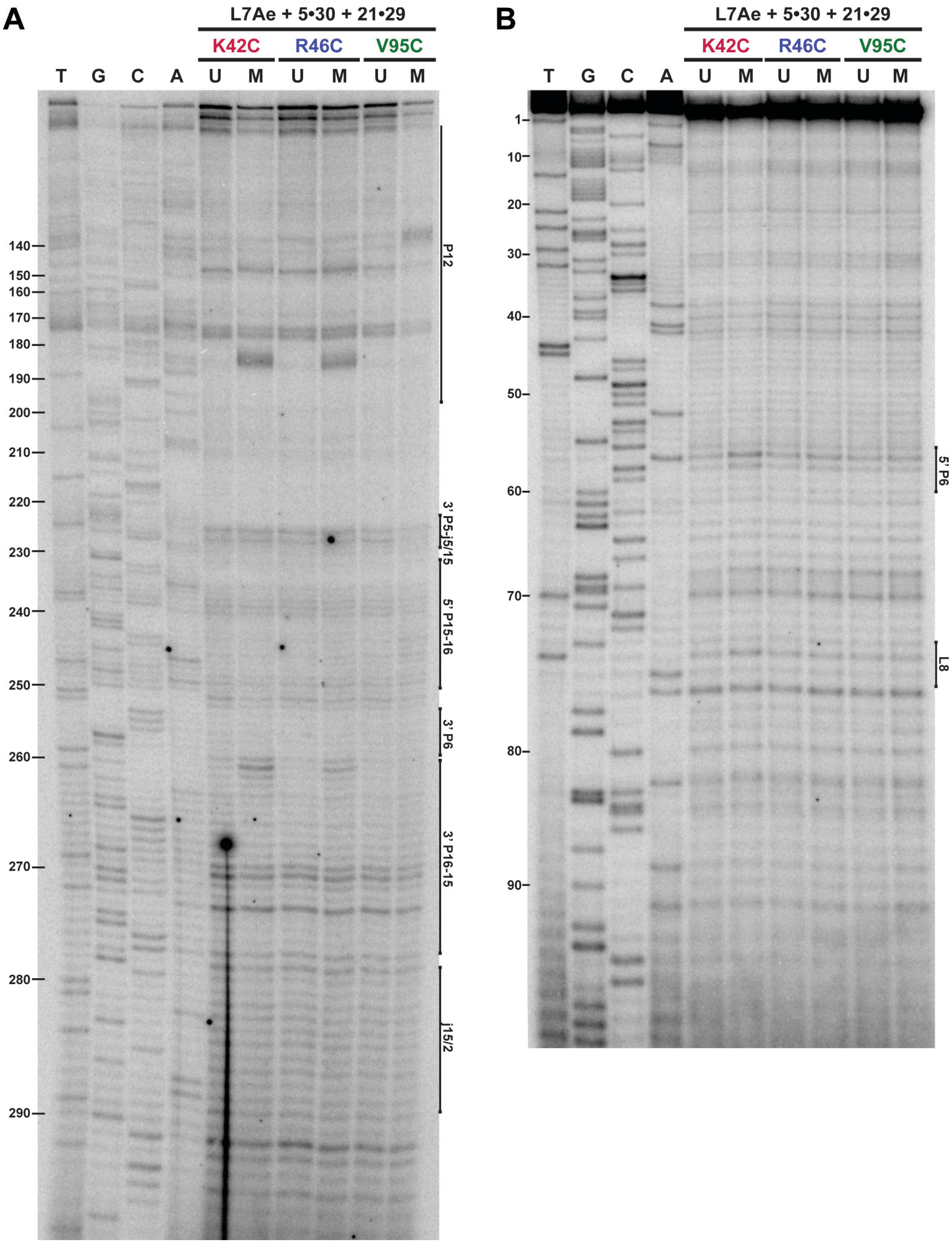

Figure S3. Mapping OH$^{\bullet}$-mediated cleavages of L7Ae–EDTA-Fe derivatives on *Pfu* RPR in the 5-RPP RNase P enzyme. RNA cleavage products were reverse transcribed using 5'-[^{32}P]-labeled primers (A) PfuRPR-1R and (B) PfuRPR-3R (Fig. 4A and Table S3). U, unmodified; M, EDTA-Fe-modified.

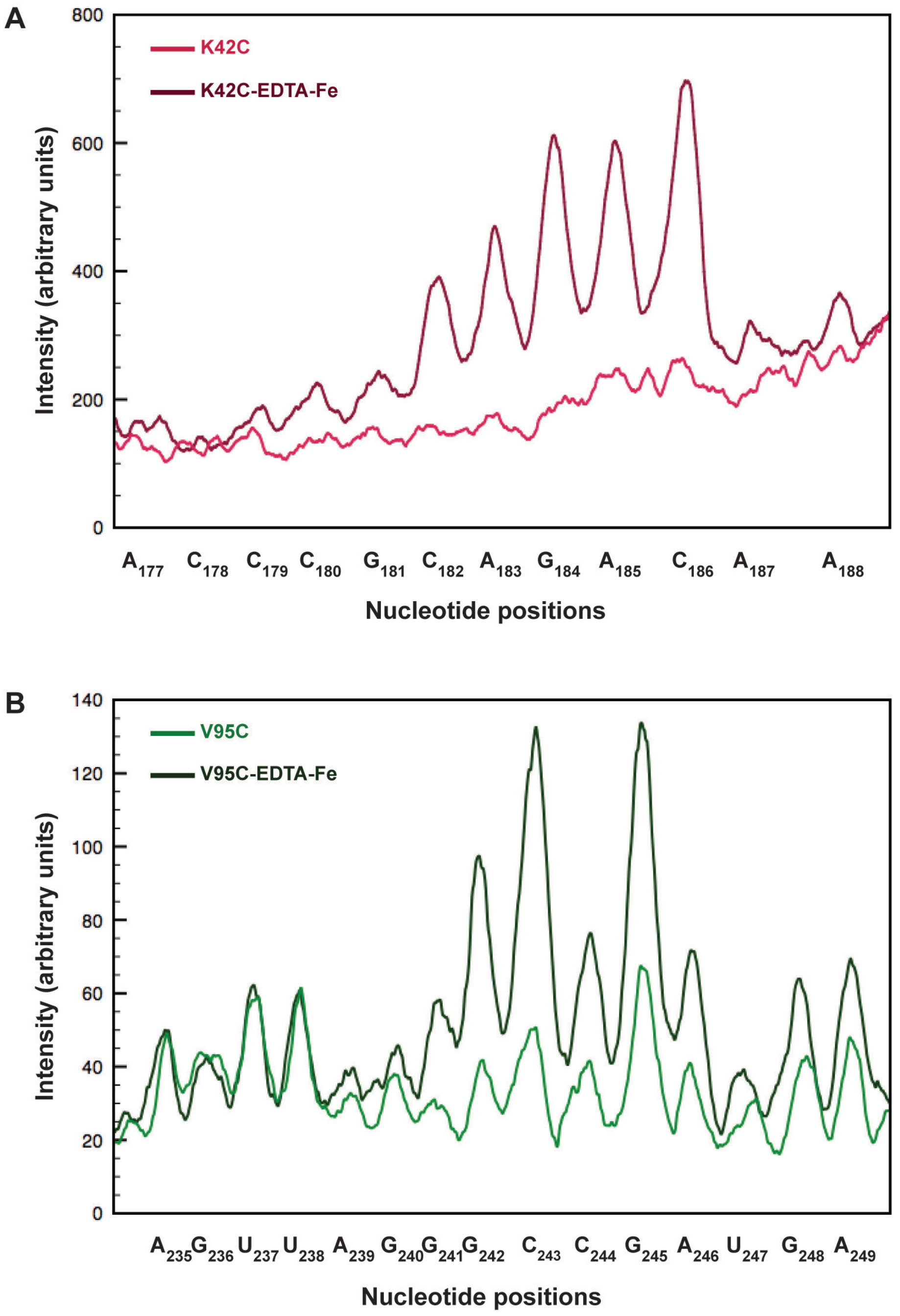

Figure S4. Representative comparisons of the intensities of OH$^{\bullet}$-mediated cleavages of *Pfu* RPR promoted by unmodified and EDTA-Fe-modified L7Ae derivatives. ImageQuant was used to quantitate individual band intensities in each reaction while Plot was used to visualize the resultant data. Each peak was identified using the sequencing ladder (x-axis) as reference; intensity is measured in arbitrary units and plotted on the y-axis. (A) Cleavages in the P12 region as promoted by K42C–EDTA-Fe. (B) Cleavages in the P16 region as promoted by V95C–EDTA-Fe.

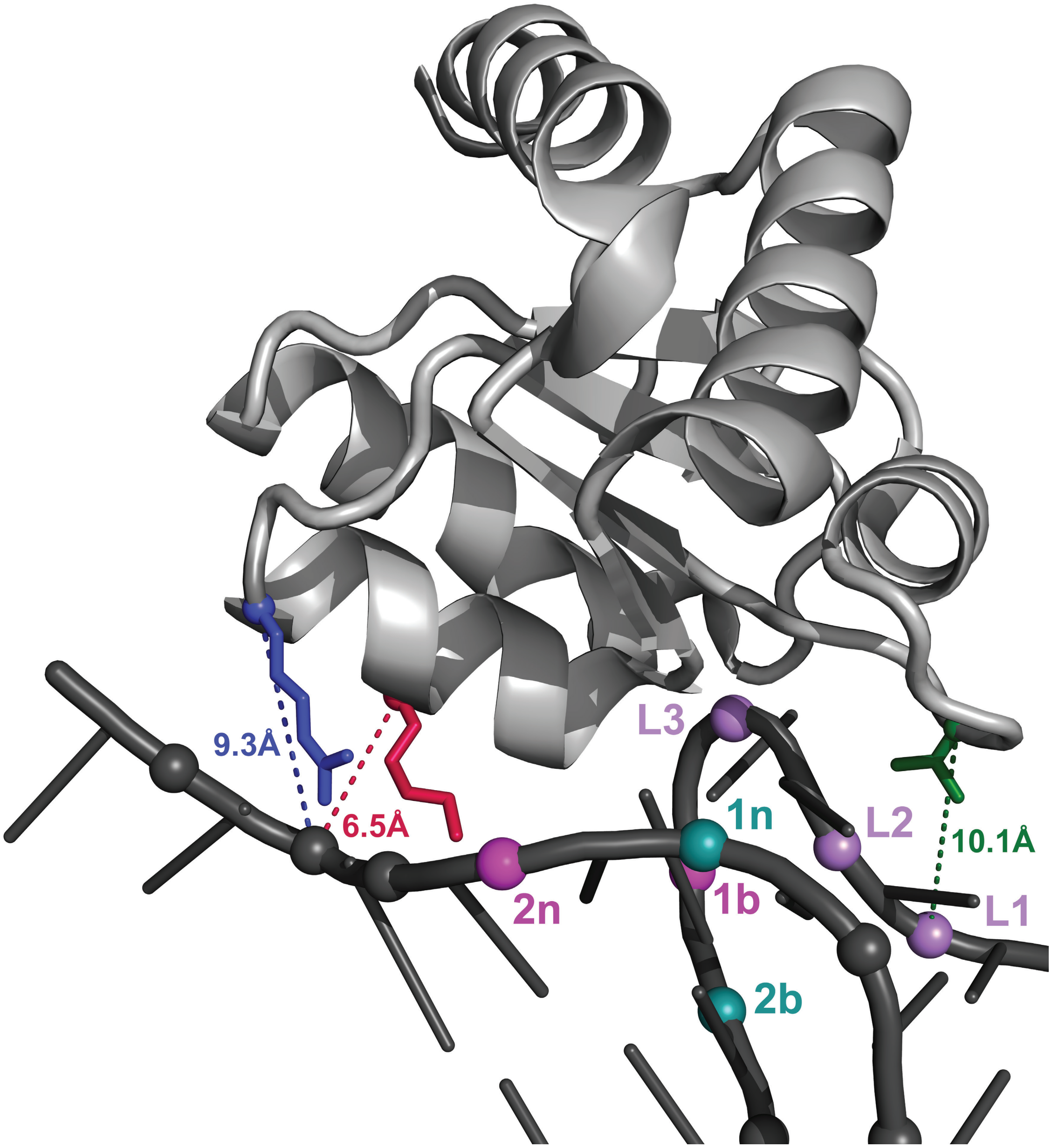

Figure S5. Representative distance measurements between K42, R46, V95 of L7Ae and proximal backbone phosphates in an RNA ligand [PDB: 3NVI (2)]. Distances were measured (in Å) from the C^{α} of K42 (red), R46 (blue), and V95 (green) to the backbone phosphates (spheres) corresponding to the most intense footprints in the P16 region for each L7Ae–EDTA-Fe reaction. The sheared G•A base pairs and three-nucleotide bulge are also labeled using the color scheme in Figure 1.

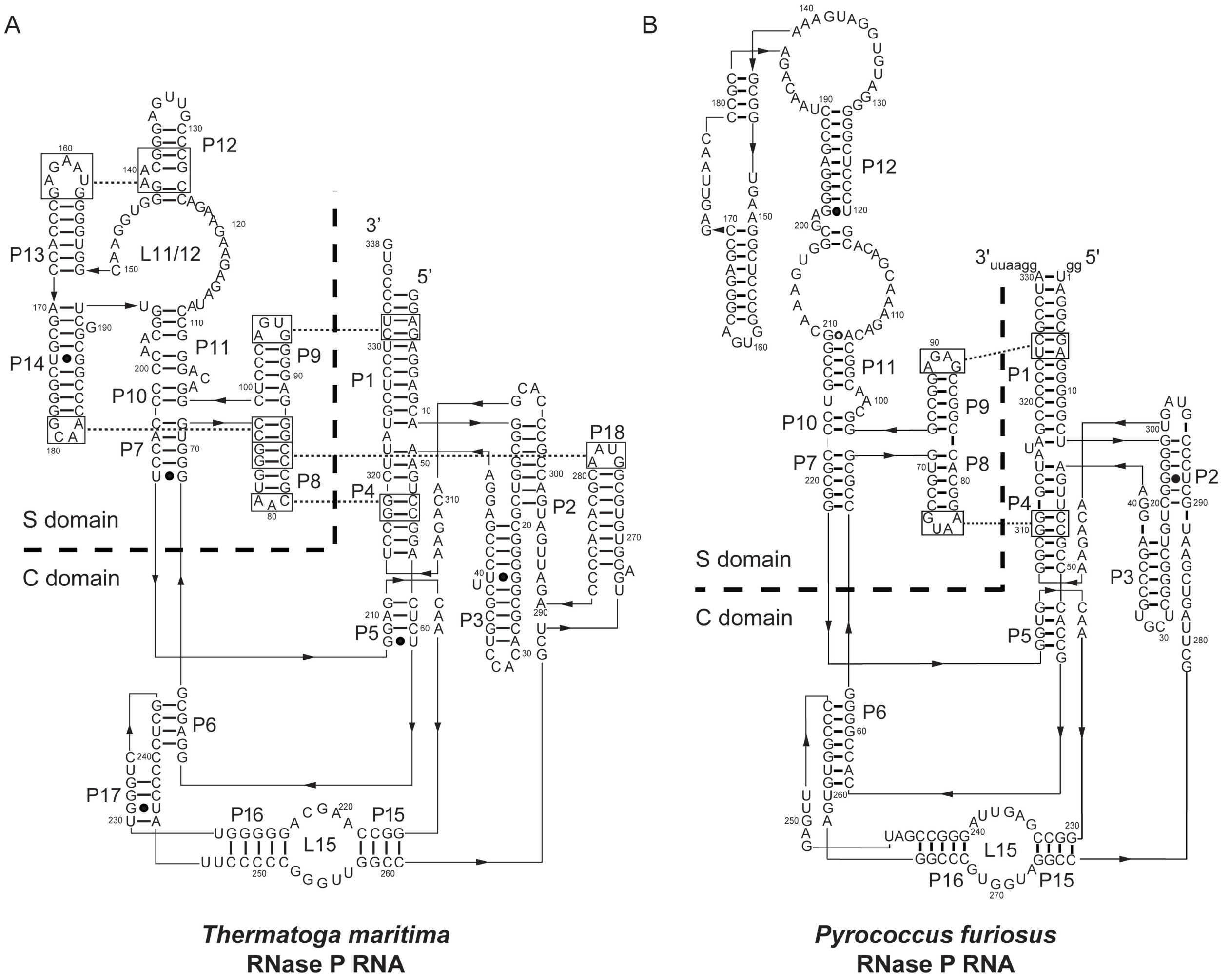

Figure S6. (A) Secondary structure of *Thermotoga maritima* (*Tma*) RNase P RNA, consistent with phylogenetic studies and high-resolution structures of bacterial RPRs (3,4). (B) Predicted secondary structure of *Pfu* RPR, modeled using as template the *Tma* secondary structure in (A).

REFERENCES

1. Smith, I.L.T. (2011) Application of Site-Directed Metal Chelation Techniques to Structure Determination of *Pyrococcus furiosus* RNase P, B.S. thesis, The Ohio State University, Columbus, OH.
2. Xue, S., Wang, R., Yang, F., Terns, R.M., Terns, M.P., Zhang, X., Maxwell, E.S. and Li, H. (2010) Structural basis for substrate placement by an archaeal box C/D ribonucleoprotein particle. *Mol. Cell*, **39**, 939-949.
3. Torres-Larios, A., Swinger, K.K., Krasilnikov, A.S., Pan, T. and Mondragón, A. (2005) Crystal structure of the RNA component of bacterial ribonuclease P. *Nature*, **437**, 584-587.
4. Brown, J.W. (1999) The Ribonuclease P Database. *Nucleic Acids Res.*, **27**, 314.
